# Development of an online prediction tool for immunotherapy-related adverse events in patients with advanced NSCLC based on machine learning and temporal validation

**DOI:** 10.3389/fonc.2026.1711801

**Published:** 2026-05-01

**Authors:** Ling-Chun Cao, Jing-Jing Ye, Wen-Qian Mei, Xue-Yan Hu, Fan-Liang Meng, Xiao-An Sheng

**Affiliations:** 1 Department of Respiratory and Critical Care Medicine, Chaohu Hospital of Anhui Medical University, Hefei, Anhui, China; 2 Department of Oncology and Radiation Therapy, Chaohu Hospital of Anhui Medical University, Hefei, Anhui, China

**Keywords:** advanced non-small cell lung cancer, immune-related adverse events, machine learning, temporal validation, web-based tool

## Abstract

**Objective:**

Despite the widespread use of immune checkpoint inhibitors (ICIs) improving survival outcomes in non-small cell lung cancer (NSCLC) patients, immune-related adverse events (irAEs) triggered by ICIs have become a major challenge in clinical practice. This study aims to establish an interpretable machine learning model to predict ICI treatment of irAE risk in advanced NSCLC patients, thereby supporting clinical decision-making and thus improving the safety of immunotherapy.

**Methods:**

A total of 550 patients were enrolled in the study. The development cohort consisted of 420 patients treated with ICIs from January 2019 to October 2023 and was randomly divided into a training set (n = 295) and a test set (n = 125). In addition, a temporally distinct cohort of 130 patients treated from November 2023 to November 2024 served as the validation set. Nine machine-learning algorithms were trained and evaluated in parallel, and the optimal model was selected based on discrimination, calibration, and clinical utility.

**Results:**

Among the 550 patients, 361 (65.6%) developed irAEs. Six essential features were chosen including neutrophil count (Neut), lymphocyte count (Lymph), platelet count (Plt), hemoglobin (Hb), Eastern Cooperative Oncology Group Performance Status (ECOG PS), and history of diabetes. Although the neural network (NN) model performed slightly better in the test set, the logistic regression (LR) model offered superior interpretability, and its clinical net benefit was similar to that of the NN model. Therefore, the LR model was ultimately selected as the optimal predictive model (AUC = 0.855 in the test set; 0.801 in the validation set).

**Conclusion:**

The LR model enables the early identification of patients at high risk of developing irAEs during hospitalization and supports their timely adoption of individualized management measures. The web tool developed based on this model is available at https://lingchun.shinyapps.io/web123/.

## 1 Introduction

Lung cancer has become a severe global challenge, significantly impacting public health and the economy. According to the latest GLOBOCAN data released by the International Agency for Research on Cancer (IARC) in 2022, there are approximately 2.5 million new cases of lung cancer annually, accounting for 12.4% of all new cancer cases. Additionally, there are about 1.8 million lung cancer-related deaths, making up 18.7% of all cancer deaths. Its incidence and mortality rates are the highest among all cancers (1). Some studies indicate that by 2050, the global annual incidence of lung cancer is expected to rise to 3.8 million cases, with corresponding mortality reaching approximately 3.2 million deaths (2). In China, lung cancer remains the principal cause of cancer-related mortality (3). Histologically, it is broadly categorized into non-small cell lung cancer (NSCLC) and small cell lung cancer (SCLC). Among them, NSCLC is the predominant pathological type, accounting for approximately 85% of all lung cancer cases (4). Currently, surgery is the primary treatment for early-stage NSCLC, while for newly diagnosed, unresectable advanced NSCLC, the emergence of immune checkpoint inhibitors (ICIs) has significantly improved patient prognosis (5, 6). Immunotherapy (including anti-programmed cell death 1 (PD-1)/programmed cell death ligand 1 (PD-L1) antibodies and anti-cytotoxic T-lymphocyte-associated protein 4 (CTLA-4) antibodies) in combination with chemotherapy has become the first-line standard treatment for advanced NSCLC (7, 8).

Immune-related adverse events (irAEs) are known to involve multiple organ systems, including the integumentary, gastrointestinal, endocrine, pulmonary, and hepatic systems, among others (9). While the majority of irAEs are mild to moderate and can be effectively controlled through treatment strategies such as pausing the medication or using corticosteroids (10), some events may progress rapidly, leading to severe or even fatal outcomes, such as severe pneumonia, severe hepatitis (11, 12). In addition, the occurrence of irAEs often requires the interruption or discontinuation of immunotherapy (13, 14). This may impact the long-term survival benefits of patients. Therefore, using risk prediction models to identify high-risk individuals for irAEs early, and implementing effective prevention and management strategies, is crucial for maximizing the clinical benefits of immunotherapy, minimizing potential risks, and ensuring patient safety and quality of life.

Recently, the development of diagnostic and prognostic models for a wide range of diseases has been substantially advanced by the application of sophisticated machine learning algorithms (15, 16). Compared to traditional statistical methods, the advantage of using machine learning algorithms to build models lies in their ability to automatically learn complex, nonlinear patterns and relationships from large datasets, thereby improving the accuracy of predictions (17–19). Moreover, some algorithms support online learning or continuous updates, allowing them to adapt to dynamic environments. However, due to the “black box” nature of machine learning algorithms, the process and logic behind model decisions are obscure, which can reduce trust in their clinical application (20, 21). The Shapley additive explanations (SHAP) method provides an effective solution to this issue by clarifying the contribution of key features and offering intuitive decision visualizations (22, 23).

This study aims to develop and validate a risk prediction model for irAEs in patients with NSCLC using machine learning algorithms combined with clinical data. Additionally, the SHAP method will be applied to conduct interpretability analysis on the best-performing model to enhance clinical understanding. Our findings from this study will help clinicians more accurately assess individual patient risks, thereby making more informed clinical decisions.

## 2 Materials and methods

### 2.1 Study subjects

This study included patients with advanced NSCLC who received ICIs at Chaohu Hospital of Anhui Medical University between January 2019 and October 2023 as the development cohort. A temporally independent validation cohort was also created from patients who got ICIs at the same facility between November 2023 and November 2024.

Relevant case data were extracted from the electronic medical record system. Inclusion criteria included: (1) Adult patients aged ≥ 18 years; (2) Pathologically diagnosed with NSCLC, with tumor-node-metastasis (TNM) staging of III-IV; (3) Completion of ≥ 2 cycles of standard ICI treatment; (4) Complete clinical, laboratory, and treatment records. Exclusion criteria included: (1) Poor patient compliance, no regular treatment or follow-up visits; (2) Pathological type of small cell lung cancer, or lack of pathological classification and early-stage (I-II) NSCLC; (3) Incomplete clinical, laboratory, and other medical records. This study has been approved by the Medical Ethics Committee of Chaohu Hospital of Anhui Medical University (KYXM-202506-003).

### 2.2 Data collection

The demographic information collected in this study included gender, age, height, weight, BMI, smoking history, family history, and comorbidities, with the latter comprising chronic obstructive pulmonary disease (COPD), coronary heart disease (CHD), hypertension, diabetes, and other comorbid medical conditions. Tumor characteristics included pathological type and TNM stage according to the AJCC (American Joint Committee on Cancer) 9th edition. Treatment information included the treatment plan, type of drug, and lines of treatment; the treatment plan was categorized as immune checkpoint inhibitor (ICI) monotherapy (I), ICI combined with chemotherapy (I + C), ICI combined with antiangiogenic therapy (I + A), and ICI combined with both antiangiogenic therapy and chemotherapy (I + A + C). The ICIs administered included camrelizumab, sintilimab, tislelizumab, pembrolizumab, and other less frequently used agents. Baseline peripheral blood biomarkers included prothrombin time (PT), activated partial thromboplastin time (APTT), thrombin time (TT), D-dimer, hemoglobin (Hb), albumin (ALB), globulin (GLB), lactate dehydrogenase (LDH), carcinoembryonic antigen (CEA), neuron-specific enolase (NSE), Cytokeratin 19 Fragment (CYFRA21-1), eosinophil count (Eos), white blood cell count (WBC), neutrophil count (Neut), lymphocyte count (Lymph), monocyte count (Mono), platelet count (Plt), alkaline phosphatase (ALP), creatinine (CREA), total bilirubin (TBIL), direct bilirubin (DBIL), indirect bilirubin (IBIL), total cholesterol (TC), triglycerides (TG), alanine aminotransferase (ALT), aspartate aminotransferase (AST), potassium (K), sodium (Na), and calcium (Ca). Moreover, the Eastern Cooperative Oncology Group Performance Status (ECOG PS) was recorded. Derived indices such as NLR (neutrophil-to-lymphocyte ratio), PLR (platelet-to-lymphocyte ratio), and LMR (lymphocyte-to-monocyte ratio) were also calculated. All baseline parameters were obtained within 1 week prior to ICI treatment.

### 2.3 irAEs assessment

The irAEs occurring during ICI treatment were standardized and assessed using the Common Terminology Criteria for Adverse Events (CTCAE 5.0) established by the National Cancer Institute (NCI). Comprehensive data were recorded, including the specific types of irAEs, severity grades, and other toxicological characteristics.

### 2.4 Feature selection and model construction

This retrospective study included variables with missing values (see **Supplementary Table 1**). All candidate variables had a missing rate of less than 20%, and therefore no variables were excluded. To minimize the influence of missingness on model development, multiple imputation based on the Fully Conditional Specification (FCS) framework was applied. The development cohort (n = 420) was randomly partitioned into a training set (n = 295) and a test set (n = 125) at a 7:3 ratios. To avoid information leakage, imputation was carried out separately within the training set, test set, and temporal external validation set. The mice package (version 3.17.0) was used to make five imputed datasets over 20 iterations with Predictive Mean Matching (PMM). Subsequently, multivariable regression models were fitted separately on each imputed dataset, and the parameter estimates and standard errors were combined using Rubin’s rules to obtain the final parameters for subsequent analyses.

**Figure 1:**
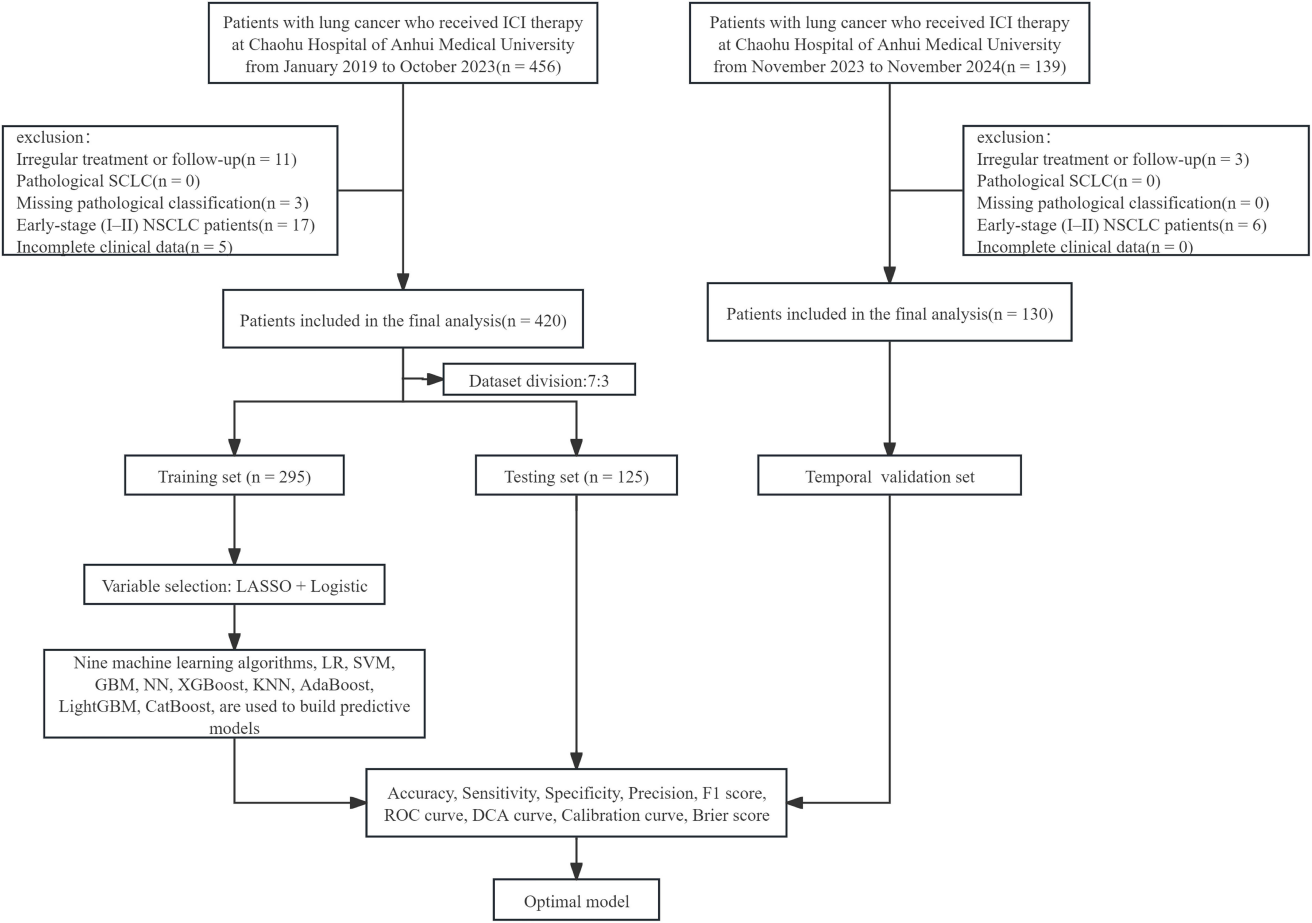
Data processing flowchart.

As illustrated in **Figure 1**, LASSO regression was applied in the feature selection stage using the training set to reduce multicollinearity and stop the model from exaggerating (24, 25). The optimal penalty coefficient (λ) was determined through 10-fold cross-validation, and a preliminary feature subset was selected based on the minimum mean squared error (MSE) criterion. These variables were then entered into multivariable logistic regression for secondary selection (inclusion criterion: P < 0.05). The final retained variables formed the predictive feature set.

In the model construction phase, nine machine learning algorithms, including Logistic Regression (LR), Support Vector Machine (SVM), Gradient Boosting Machine (GBM), Neural Network (NN), Extreme Gradient Boosting (XGBoost), K-Nearest Neighbors (KNN), Adaptive Boosting (AdaBoost), Light Gradient Boosting Machine (LightGBM), and Categorical Boosting (CatBoost), were used to construct predictive models. The optimal hyperparameter configurations for each model were determined within the training set using grid search combined with five-times repeated 10-fold cross-validation (26). On this basis, we conducted the final unbiased performance evaluation using the test set and further assessed the generalizability of the models through an external validation cohort. Evaluation metrics included accuracy, sensitivity, specificity, precision, and F1 score, all calculated from the confusion matrix (27) (see **Supplementary Figures 1**, **2**). Furthermore, the model’s discriminatory ability, clinical utility, and calibration were assessed using the receiver operating characteristic (ROC) curve, decision curve analysis (DCA), Brier score, and calibration curve.

### 2.5 SHAP explanation and web deployment

The optimal model utilizes SHAP values to visualize feature importance and is deployed as an interactive web application developed with the R Shiny framework (28), making the prediction process more aligned with clinical needs.

### 2.6 Statistical methods

In this study, baseline data were analyzed using SPSS 27.0. Continuous variables with a normal distribution are presented as mean ± standard deviation (mean ± SD), while those with a non-normal distribution are presented as median (interquartile range) [M(Q1, Q3)]. Categorical data are presented as frequency (percentage) [n (%)]. Group comparisons were conducted using t-tests, Wilcoxon rank-sum tests, Fisher’s exact tests, or Chi-square tests based on the data type and distribution characteristics. A two-tailed p-value of P < 0.05 was considered statistically significant. Model training was performed using R version 4.5.0 and the caret package (version 7.0-1). SHAP interpretation was performed using the shapviz package (version 0.9.7), and web deployment was implemented using the shiny package (version 1.10.0).

## 3 Results

### 3.1 Comparison of baseline data between the irAEs and the non-irAEs groups in the development cohort and training set

After screening based on the inclusion and exclusion criteria, a total of 420 advanced non-small cell lung cancer patients who received at least two cycles of ICI therapy were included in the development cohort. In addition, 130 patients treated between November 2023 and November 2024 were included as an independent temporal validation cohort. Patients were divided into the irAEs and the non-irAEs groups based on whether immune-related adverse events occurred. As shown in **Table 1**, in the development cohort, the irAEs group had significantly higher height, BMI and weight compared to the non-irAEs group, with statistical significance (P < 0.05). Regarding medical history, the proportion of patients in the irAEs group with ECOG PS ≥ 2 and a history of diabetes was significantly higher than in the non-irAEs group, with statistical significance (P < 0.05). In terms of laboratory data, the irAEs group had significantly higher levels of LMR, Hb, Eos, Lymph, and Na, while NLR, PLR, D-dimer, GLB, CYFRA21-1, WBC, Neut, and Plt levels were significantly lower than those in the non-irAEs group, with statistical significance (P < 0.05). As shown in **Table 2**, in the training set, the irAEs group had significantly higher height, weight, and BMI compared to the non-irAEs group, with statistical significance (P < 0.05). Regarding medical history, the proportion of patients in the irAEs group with ECOG PS ≥ 2 and a history of diabetes was significantly higher than in the non-irAEs group, with statistical significance (P < 0.05). In terms of laboratory data, the irAEs group had significantly higher levels of LMR, Hb, Eos, Lymph, ALT, and Na, while NLR, PLR, D-dimer, GLB, CYFRA21-1, WBC, Neut, and Plt levels were significantly lower than those in the non-irAEs group, with statistical significance (P < 0.05).

**Table 1:** Comparison of baseline data between the irAEs and non-irAEs groups in the development cohort [n (%), mean ± SD, M (Q1, Q3)].

Variable	irAEs group (n = 279)	non-irAEs group (n = 141)	Z/t/χ2	p
Gender			0.060	0.806
Female	41 (15)	22 (16)		
Male	238 (85)	119 (84)		
Family history				
No	277 (99)	138 (98)	0.612	0.434
Yes	2 (1)	3 (2)		
Smoking history			0.036	0.850
No	161 (58)	80 (57)		
Yes	118 (42)	61 (43)		
Hypertension			2.334	0.127
No	177 (63)	100 (71)		
Yes	102 (37)	41 (29)		
Diabetes			8.301	0.004
No	245 (88)	136 (96)		
Yes	34 (12)	5 (4)		
COPD			0.013	0.980
No	269 (96)	137 (97)		
Yes	10 (4)	4 (3)		
CHD			1.224	0.269
No	262 (94)	136 (96)		
Yes	17 (6)	5 (4)		
Comorbidities			3.322	0.068
No	138 (49)	83 (59)		
Yes	141 (51)	58 (41)		
Pathological type			2.141	0.337
LUAD	110 (39)	53 (38)		
SQCC	161 (58)	87 (62)		
Others	8 (3)	1 (1)		
T stage			3.769	0.438
T1	32 (11)	11 (8)		
T2	67 (24)	36 (26)		
T3	50 (18)	23 (16)		
T4	101 (36)	61 (43)		
Tx	29 (10)	10 (7)		
N stage			4.402	0.354
N0	29 (10)	24 (17)		
N1	30 (11)	11 (8)		
N2	134 (48)	64 (45)		
N3	68 (24)	32 (23)		
Nx	18 (6)	10 (7)		
M stage			1.184	0.227
M0	102 (37)	44 (31)		
M1	177 (63)	97 (69)		
Treatment plan			0.680	0.878
I	52 (19)	22 (16)		
I + C	165 (59)	88 (62)		
I + A	33 (12)	16 (11)		
I + A + C	29 (10)	15 (11)		
Type of drug,			1.281	0.865
Camrelizumab	86 (31)	45 (32)		
Sintilimab	64 (23)	38 (27)		
Tislelizumab	79 (28)	35 (25)		
Pembrolizumab	32 (11)	14 (10)		
Others	18 (6)	9 (6)		
Lines of treatment			0.222	0.911
1	235 (84)	121 (86)		
2	36 (13)	17 (12)		
≥ 3	8 (3)	3 (2)		
ECOG PS			11.640	0.003
0	32 (11)	22 (16)		
1	184 (66)	106 (75)		
≥ 2	63 (23)	13 (9)		
Age (years)	68 (60, 73)	67 (63, 72)	-0.269	0.788
Height (cm)	167 (162, 171)	165 (160, 170)	-2.484	0.013
Weight (kg)	61 (55, 70)	58 (51, 65)	-3.585	< 0.001
BMI (kg/m^2^)	22.27 (20.11, 24.57)	21.26 (19.57, 23.14)	-2.837	0.005
NLR	2.81 (2.05, 3.74)	4.95 (3.51, 7)	-9.235	< 0.001
PLR	142.02 (106.84, 184.86)	231.15 (174.51, 301.18)	-9.622	< 0.001
LMR	2.78 (1.96, 3.65)	2.1 (1.43, 2.91)	-5.313	< 0.001
PT(s)	12.5 (11.4, 13.2)	12.5 (11.4, 13.4)	-0.799	0.424
APTT(s)	32.6 (26.5, 36.9)	28.9 (26, 36.7)	-1.569	0.117
TT(s)	17.1 (16.35, 18)	17 (16.1, 17.7)	-1.308	0.191
D-dimer(μg/ml)	0.61 (0.36, 1.15)	0.78 (0.45, 1.74)	-3.137	0.002
Hb(g/L)	122.98 ± 16.73	115.63 ± 17.15	-4.218	< 0.001
ALB(g/L)	37 (34, 39.6)	36.2 (33.5, 38.9)	-1.766	0.078
GLB(g/L)	28.2 (25.1, 31.75)	30 (25.8, 34.5)	-2.624	0.009
LDH(U/L)	186 (161, 228.5)	196 (163, 245)	-1.520	0.129
CEA(ng/ml)	4.05 (2.52, 10.39)	4.86 (2.42, 15.01)	-0.805	0.421
NSE(ng/ml)	15.1 (12.8, 18.74)	16 (12.2, 21.6)	-0.781	0.435
CYFRA21-1(ng/ml)	5.18 (2.86, 11.4)	6.88 (3.64, 18.3)	-2.814	0.005
Eos(×10^9^/L)	0.14 (0.08, 0.23)	0.09 (0.03, 0.21)	-3.717	< 0.001
WBC(×10^9^/L)	6.11 (5.11, 7.6)	7.29 (5.34, 9.57)	-4.101	< 0.001
Neut (×10^9^/L)	3.87 (3.04, 5.02)	5.51 (3.86, 7.46)	-6.454	< 0.001
Lymph(×10^9^/L)	1.4 (1.09, 1.78)	1.12 (0.87, 1.4)	-5.562	< 0.001
Mono(×10^9^/L)	0.49 (0.4, 0.63)	0.55 (0.4, 0.79)	-1.739	0.082
Plt(×10^9^/L)	199 (153.5, 247)	256 (200, 321)	-6.254	< 0.001
ALP(U/L)	87 (71.5, 107.5)	91 (73, 114)	-0.947	0.344
CREA(μmol/L)	63 (53.5, 74)	62 (52, 72)	-1.001	0.317
TC(mmol/L)	4.32 (3.74, 5.03)	4.29 (3.61, 5.24)	-0.165	0.869
TG(mmol/L)	1.05 (0.84, 1.35)	1.02 (0.77, 1.32)	-0.952	0.341
TBIL(μmol/L)	10.5 (8.15, 13.65)	10 (8.1, 13)	-0.892	0.372
DBIL(μmol/L)	2.4 (1.7, 3.4)	2.4 (1.7, 3.3)	-0.198	0.843
IBIL(μmol/L)	8 (6.1, 10.6)	7.6 (6, 10.1)	-1.190	0.234
ALT(U/L)	17 (12, 25)	15 (11, 22)	-1.760	0.079
AST(U/L)	21 (17, 26)	19 (16, 25)	-1.502	0.133
K(mmol/L)	4.12 (3.86, 4.36)	4.15 (3.9, 4.4)	-0.868	0.386
Na(mmol/L)	140.3 (138.8, 141.8)	139.6 (137.6, 141.2)	-2.793	0.005
Ca(mmol/L)	2.26 (2.17, 2.33)	2.23 (2.13, 2.34)	-1.663	0.096

ICI, immune checkpoint inhibitor; I, ICI monotherapy; I + C, ICI + chemotherapy; I + A, ICI + antiangiogenic therapy; I + A + C, ICI + antiangiogenic therapy + chemotherapy.

**Table 2:** Comparison of baseline data between the irAEs and non-irAEs groups in the training set [n (%), mean ± SD, M (Q1, Q3)].

Variable	irAEs group (n=196)	non-irAEs group (n=99)	Z/t/χ2	p
Gender			0.023	0.879
Female	27 (14)	13 (13)		
Male	169 (86)	86 (87)		
Family history			Fisher	0.604
No	194 (99)	97 (98)		
Yes	2 (1)	2 (2)		
Smoking history			0.030	0.862
No	109 (56)	54 (55)		
Yes	87 (44)	45 (45)		
Hypertension			0.861	0.173
No	125 (64)	71 (72)		
Yes	71 (36)	28 (28)		
Diabetes			5.636	0.018
No	171 (87)	95 (96)		
Yes	25 (13)	4 (4)		
COPD			0.000	1.000
No	190 (97)	96 (97)		
Yes	6 (3)	3 (3)		
CHD			2.453	0.117
No	181 (92)	96 (97)		
Yes	15 (8)	3 (3)		
Comorbidities			2.973	0.085
No	96 (49)	59 (60)		
Yes	100 (51)	40 (40)		
Pathological type			0.352	0.900
LUAD	75 (38)	37 (37)		
SQCC	117 (60)	61 (62)		
Others	4 (2)	1 (1)		
T stage			3.777	0.437
T1	27 (14)	7 (7)		
T2	44 (22)	22 (22)		
T3	36 (18)	18 (18)		
T4	73 (37)	45 (45)		
Tx	16 (8)	7 (7)		
N stage			7.406	0.116
N0	18 (9)	19 (19)		
N1	20 (10)	7 (7)		
N2	93 (47)	46 (46)		
N3	53 (27)	24 (24)		
Nx	12 (6)	3 (3)		
M stage			0.566	0.452
M0	70 (36)	31 (31)		
M1	126 (64)	68 (69)		
Treatment plan			4.995	0.172
I	41 (21)	11 (11)		
I + C	116 (59)	63 (64)		
I + A	23 (12)	13 (13)		
I + A + C	16 (8)	12 (12)		
Type of drug			1.144	0.887
Camrelizumab	55 (28)	33 (33)		
Sintilimab	48 (24)	23 (23)		
Tislelizumab	57 (29)	28 (28)		
Pembrolizumab	20 (10)	9 (9)		
Others	16 (8)	6 (6)		
Lines of treatment			2.048	0.376
1	159 (81)	87 (88)		
2	29 (15)	10 (10)		
≥ 3	8 (4)	2 (2)		
ECOG PS			6.674	0.036
0	20 (10)	15 (15)		
1	131 (67)	73 (74)		
≥ 2	45 (23)	11 (11)		
Age(years)	67.5 (60, 72.25)	68 (63, 73)	-0.765	0.445
Height(cm)	168 (161, 172)	165 (160, 170)	-2.448	0.014
Weight(kg)	61 (55, 70)	58 (51.5, 65)	-3.187	< 0.001
BMI(kg/m^2^)	22.48 ± 3.15	21.39 ± 2.94	-2.868	0.004
NLR	2.8 (2.08, 3.83)	5.03 (3.75, 6.88)	-7.782	< 0.001
PLR	144.2 (110.92, 188.06)	227.75 (174.83, 293.4)	-7.589	< 0.001
LMR	2.86 (1.9, 3.69)	2.13 (1.52, 2.94)	-4.024	< 0.001
PT(s)	12.4 (11.4, 13.2)	12.4 (11.45, 13.25)	-0.147	0.883
APTT(s)	31.85 (26.4, 36.8)	28.8 (25.9, 36.65)	-1.380	0.168
TT(s)	17.1 (16.4, 18.02)	17.1 (16.15, 17.9)	-1.100	0.271
D-dimer(μg/ml)	0.6 (0.34, 1.23)	0.78 (0.42, 1.62)	-2.634	0.008
Hb(g/L)	123.16 ± 16.44	114.7 ± 18.58	-3.996	< 0.001
ALB(g/L)	36.85 (33.98, 39.6)	35.6 (33.1, 38.9)	-1.366	0.172
GLB(g/L)	28.65 ± 5.30	30.36 ± 5.47	-2.579	0.010
LDH(U/L)	185 (163, 229)	199 (164.5, 248)	-1.733	0.083
CEA(ng/ml)	4.06 (2.54, 11.06)	5 (2.43, 22.2)	-0.658	0.511
NSE(ng/ml)	15.1 (12.78, 18.53)	16.28 (12.8, 21.75)	-1.494	0.135
CYFRA21-1(ng/ml)	5.24 (2.91, 11.67)	6.97 (3.98, 16.7)	-2.592	0.010
Eos(×10^9^/L)	0.14 (0.09, 0.23)	0.08 (0.03, 0.2)	-3.406	< 0.001
WBC(×10^9^/L)	6.16 (5.13, 7.69)	7.46 (5.59, 9.63)	-3.854	< 0.001
Neut(×10^9^/L)	3.92 (3.12, 5.05)	5.6 (4.09, 7.52)	-5.975	< 0.001
Lymph(×10^9^/L)	1.42 (1.09, 1.81)	1.14 (0.88, 1.43)	-4.382	< 0.001
Mono(×10^9^/L)	0.49 (0.4, 0.65)	0.56 (0.38, 0.78)	-1.524	0.128
Plt(×10^9^/L)	209 (165.25, 252.75)	257 (200.5, 321)	-4.858	< 0.001
ALP(U/L)	85.5 (72, 105.25)	92 (73.5, 111)	-1.125	0.261
CREA(μmol/L)	62 (54, 73)	60 (51.5, 73.5)	-0.669	0.504
TC(mmol/L)	4.2 (3.66, 4.9)	4.31 (3.62, 5.12)	-0.524	0.601
TG(mmol/L)	1 (0.8, 1.33)	1.02 (0.8, 1.32)	-0.198	0.844
TBIL(μmol/L)	10.5 (8.07, 13.62)	10.3 (8.15, 13.1)	-0.297	0.767
DBIL(μmol/L)	2.4 (1.7, 3.7)	2.6 (1.8, 3.6)	-0.531	0.596
IBIL(μmol/L)	7.9 (6, 10.77)	7.6 (6, 10.1)	-0.890	0.374
ALT(U/L)	17 (13, 25)	14 (10.5, 21)	-2.563	0.010
AST(U/L)	21 (17.75, 26)	19 (15.5, 26.5)	-1.454	0.146
K(mmol/L)	4.18 ± 0.43	4.09 ± 0.40	1.681	0.094
Na(mmol/L)	140.3 (138.78, 141.8)	139.4 (137.4, 140.7)	-3.415	< 0.001
Ca(mmol/L)	2.22 (2.13, 2.33)	2.26 (2.17, 2.33)	-1.639	0.101

ICI, immune checkpoint inhibitor; I, ICI monotherapy; I + C, ICI + chemotherapy; I + A, ICI + antiangiogenic therapy; I + A + C, ICI + antiangiogenic therapy + chemotherapy.

### 3.2 Immune-related adverse events

**Figure 2:**
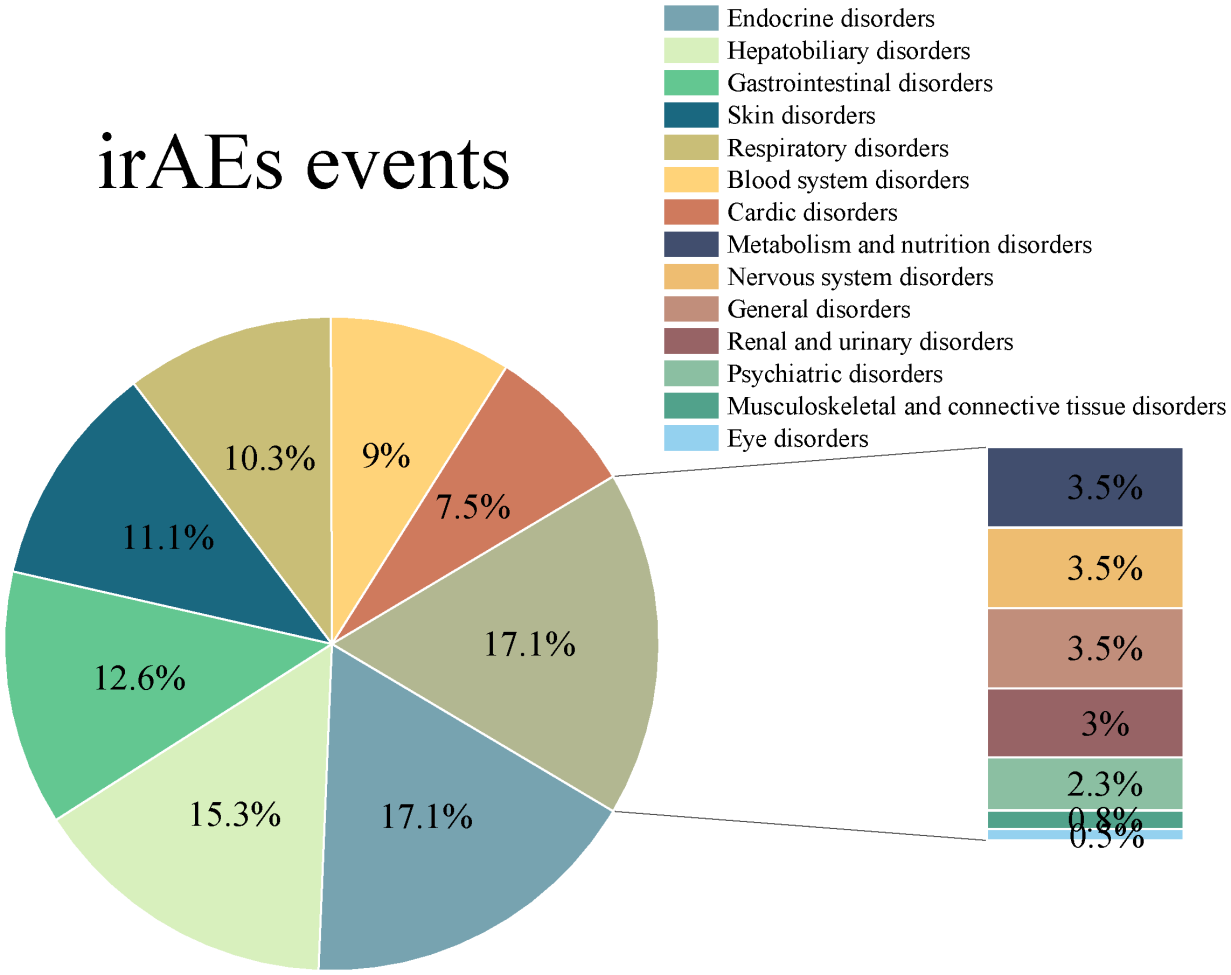
Distribution characteristics of irAEs events.

As shown in **Figure 2** and **Table 3**, a total of 361 patients in this study experienced 398 instances of any-grade irAEs, of which 55 were grade ≥3 events. Endocrine disorders were the most common irAEs, with an incidence of 17.1%, followed by hepatobiliary disorders (15.3%), gastrointestinal disorders (12.6%), skin disorders (11.1%), and respiratory disorders (10.3%). In contrast, respiratory and skin disorders were more severe, accounting for 32.7% and 23.6% of all grade ≥3 irAEs, respectively.

**Table 3:** Classification and grading of immune-related adverse events.

irAEs events	Any grade (n = 398)	Grade ≥ 3 (n = 55)
Endocrine disorders	68 (17.1)	2 (3.7)
Hepatobiliary disorders	61 (15.3)	6 (10.9)
Gastrointestinal disorders	50 (12.6)	7 (12.7)
Skin disorders	44 (11.1)	13 (23.6)
Respiratory disorders	41 (10.3)	18 (32.7)
Blood system disorders	36 (9.0)	2 (3.6)
Cardic disorders	30 (7.5)	3 (5.5)
Metabolism and nutrition disorders	14 (3.5)	0 (0)
Nervous system disorders	14 (3.5)	2 (3.7)
General disorders	14 (3.5)	0 (0)
Renal and urinary disorders	12 (3.0)	1 (1.8)
Psychiatric disorders	9 (2.3)	0 (0)
Musculoskeletal and connective tissue disorders	3 (0.8)	0 (0)
Eye disorders	2 (0.5)	1 (1.8)

### 3.3 Variable selection

**Figure 3:**
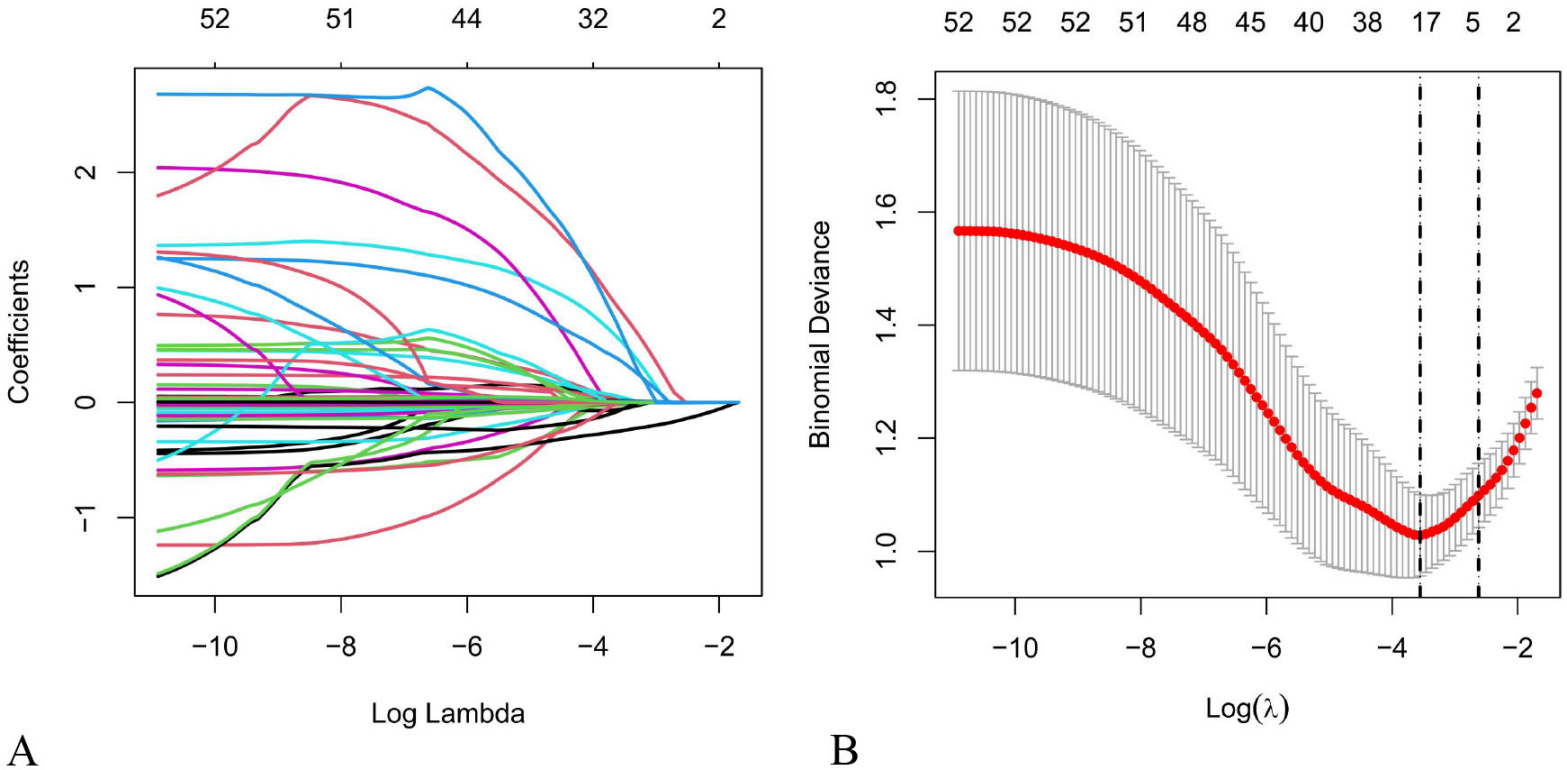
**(A)** Coefficient path plot **(B)** cross-validation plot.

**Figure 4:**
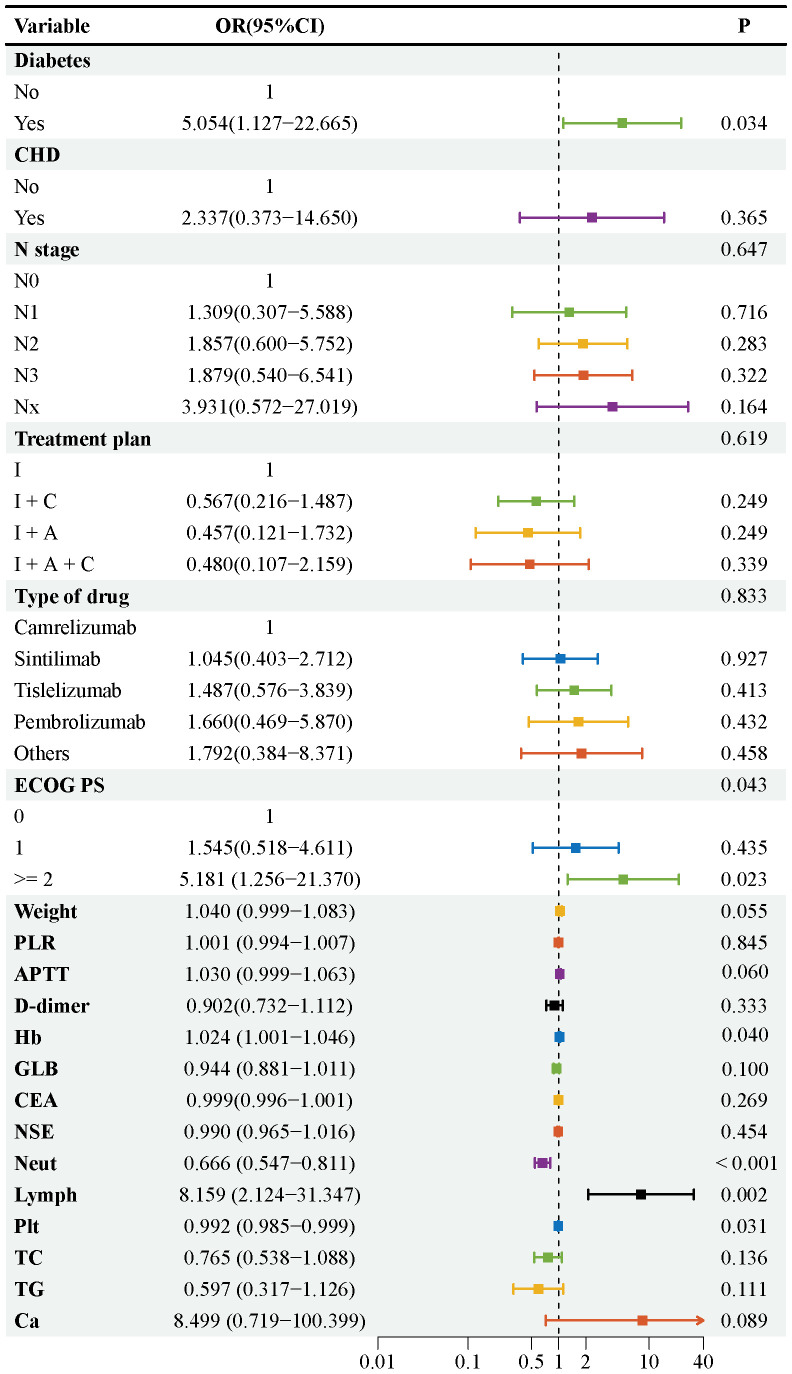
Forest plot of multivariate logistic regression results. The squares represent the adjusted ORs, odds ratios, the horizontal lines indicate 95% confidence intervals (95% CIs), and the vertical dashed line represents the invalid line (OR = 1), which is the reference line unrelated to the outcome. Variables located on the right side of the reference line (OR > 1) suggest an increased risk of irAEs, while the variables located on the left side (OR < 1) suggest a decreased risk of irAEs. For categorical variables, estimates were calculated relative to the corresponding reference categories: no diabetes, no CHD, N0 stage, immune checkpoint inhibitor monotherapy, camrelizumab treatment, and ECOG performance status 0. Continuous variables were included in the model in a continuous form, and the P-values of each variable are listed on the right side of the graph.

In the training set, this study used LASSO regression with 10-fold cross-validation for feature selection (**Figure 3**), determining the optimal penalty coefficient (λ = 0.0286), and selecting 20 non-zero coefficient variables (Diabetes, CHD, N, Treatment plan, Type of drug, ECOG PS, Weight, PLR, APTT, D-dimer, Hb, GLB, CEA, NSE, Neut, Lymph, Plt, TC, TG, and Ca). Multivariate logistic regression analysis further identified 6 independent risk factors, including a history of diabetes [p = 0.034; OR (95% CI) = 5.054 (1.127, 22.665)], ECOG PS ≥ 2 [p = 0.023; OR (95% CI) = 5.181 (1.256, 21.370)], Hb [p = 0.040; OR (95% CI) = 1.024 (1.001, 1.046)], Neut [P < 0.001; OR (95% CI) = 0.666 (0.547, 0.811)], Lymph [p = 0.002; OR (95% CI) = 8.159 (2.124, 31.347)], and Plt [p = 0.031; OR (95% CI) = 0.992 (0.985, 0.999)] (**Figure 4**).

### 3.4 Model construction and performance evaluation

**Figure 5:**
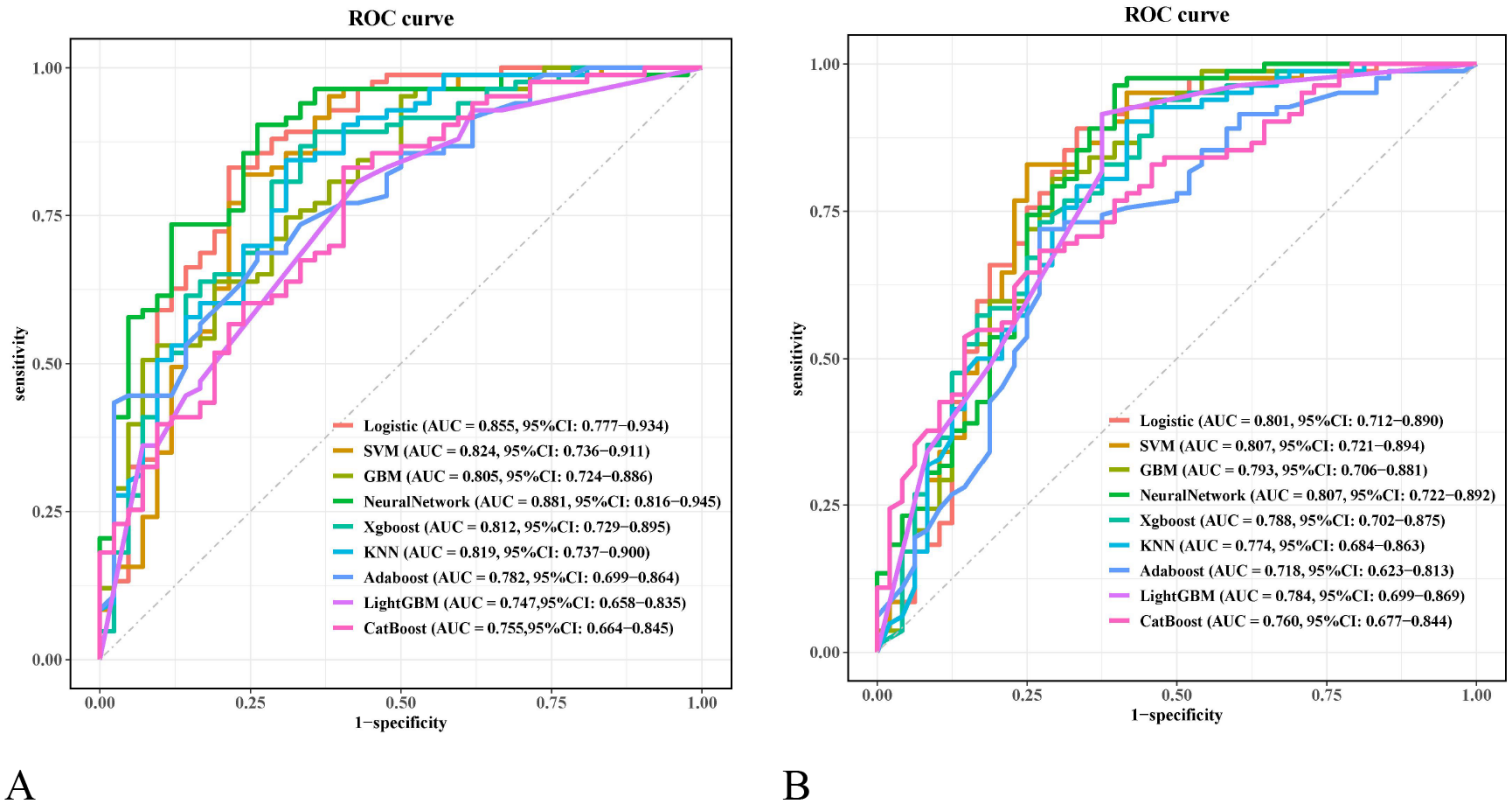
**(A)** ROC curves of nine machine learning models in the test set. **(B)** ROC curves of nine machine learning models in the validation set.

**Figure 6:**
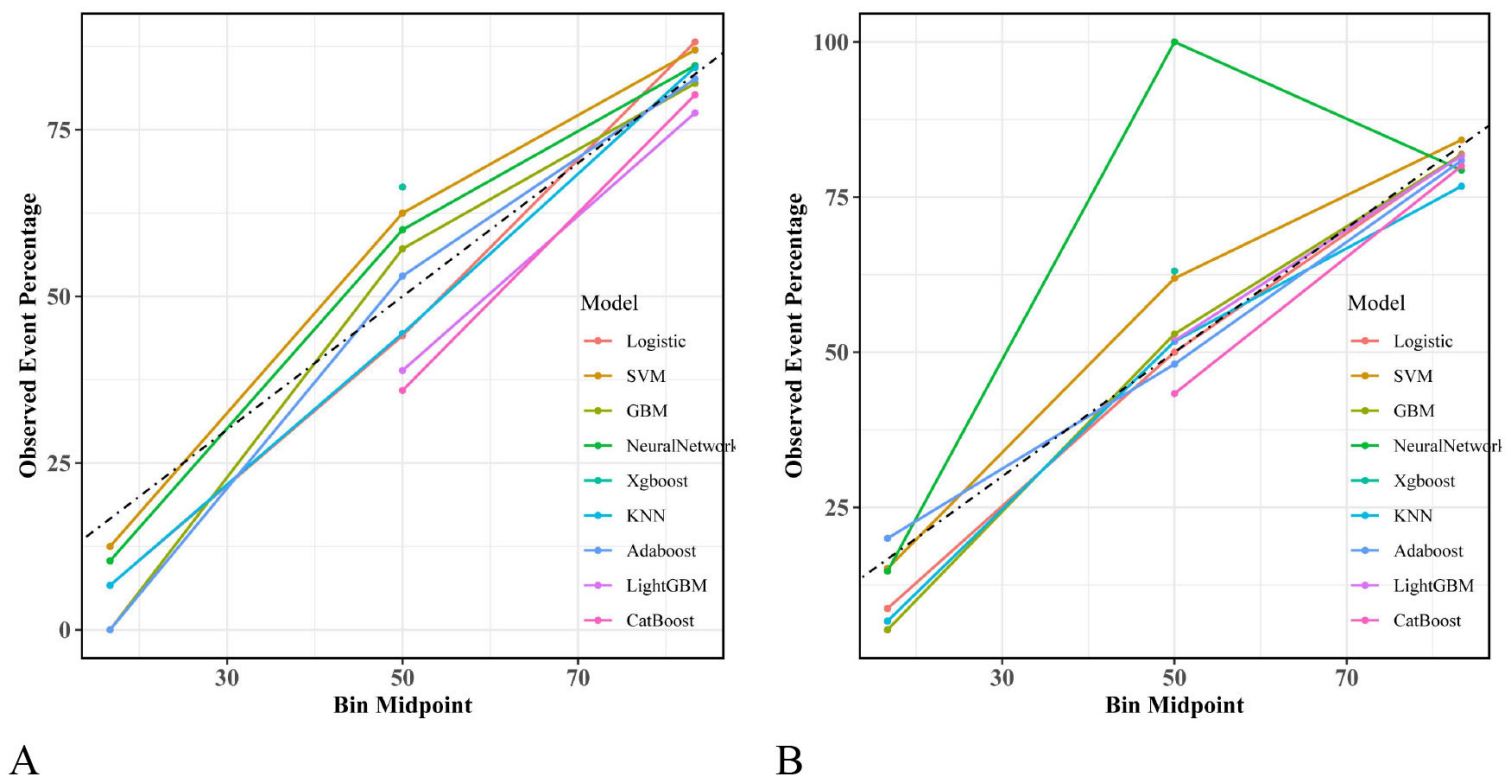
**(A)** Calibration curves of nine machine learning models in the test set. **(B)** Calibration curves of nine machine learning models in the validation set.

**Figure 7:**
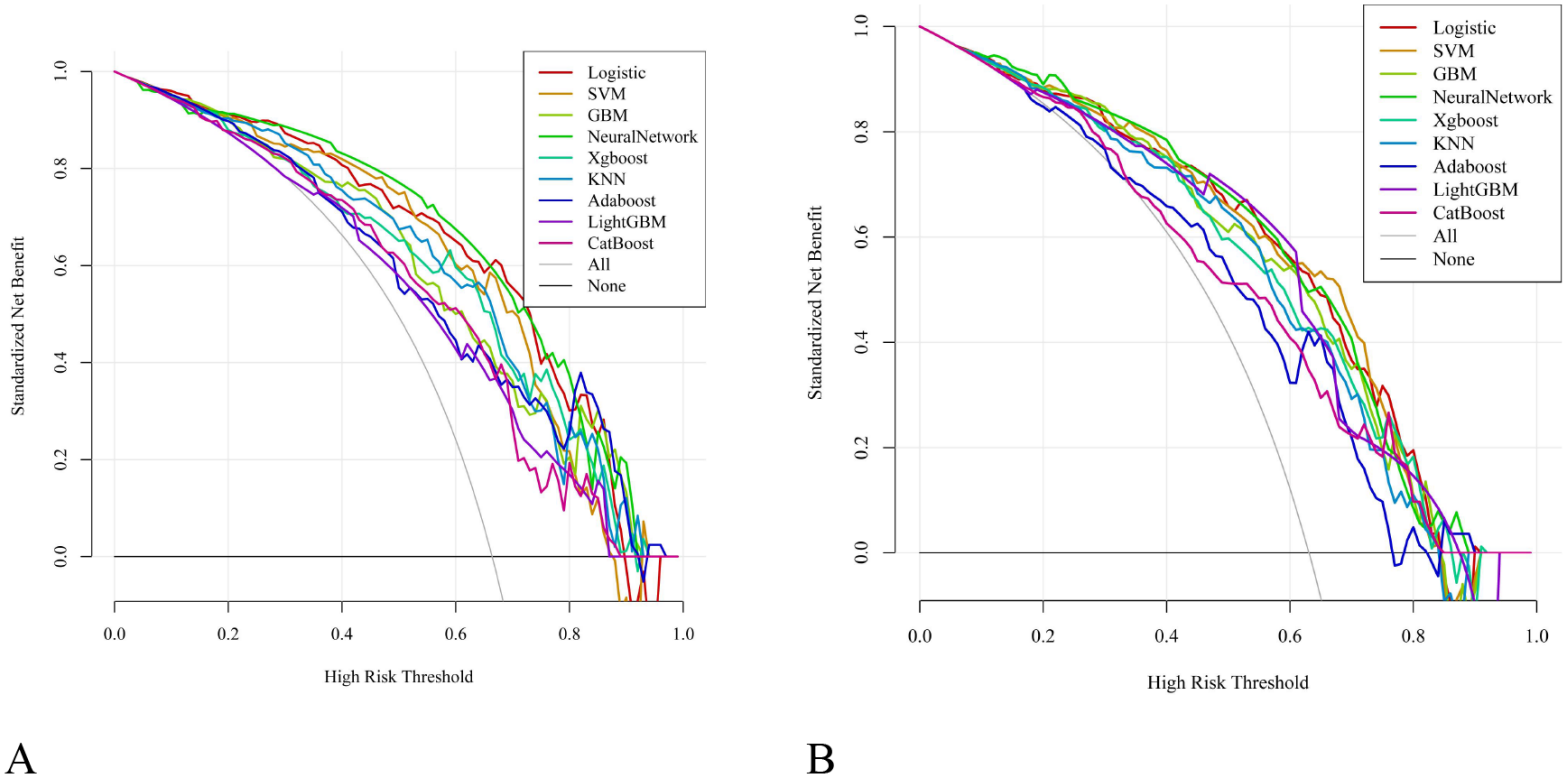
**(A)** Decision curve analysis of nine machine learning models in the test set. **(B)** Decision curve analysis of nine machine learning models in the validation set.

Based on the six independent risk factors mentioned above, this study constructed irAEs prediction models using nine machine-learning algorithms. Model performance was determined by ten-fold cross-validation. **Figures 5A, B** show the ROC performance of the nine ML methods—LR, SVM, GBM, NN, XGBoost, KNN, AdaBoost, LightGBM, and CatBoost—on the test set and external validation set, respectively. In the test set, the predictive performance of the nine algorithms is illustrated by their ROC curves (**Figure 5A**), with AUC values (95% CI) of 0.855 (0.777, 0.934), 0.824 (0.736, 0.911), 0.805 (0.724, 0.886), 0.881 (0.816, 0.945), 0.812 (0.729, 0.895), 0.819 (0.737, 0.900), 0.782 (0.699, 0.864), 0.747 (0.658, 0.835), and 0.755 (0.664, 0.845), respectively. In the external validation set, the corresponding ROC curves are shown in **Figure 5B**, with AUC values (95% CI) of 0.801 (0.712, 0.890), 0.807 (0.721, 0.894), 0.793 (0.706, 0.881), 0.807 (0.722, 0.892), 0.788 (0.702, 0.875), 0.774 (0.684, 0.863), 0.718 (0.623, 0.813), 0.784 (0.699, 0.869), and 0.760 (0.677, 0.844), respectively. **Table 4** shows the detailed performance metrics for both datasets. The F1 scores in the test set were 0.857, 0.845, 0.863, 0.888, 0.860, 0.843, 0.755, 0.798, and 0.817, while those in the external validation set were 0.854, 0.840, 0.815, 0.878, 0.844, 0.841, 0.766, 0.857, and 0.742, respectively. The Brier scores in the test set were 0.134, 0.145, 0.159, 0.123, 0.207, 0.153, 0.174, 0.209, and 0.208, and in the external validation set were 0.160, 0.158, 0.165, 0.151, 0.211, 0.177, 0.199, and 0.227, respectively. Calibration curves showed good calibration consistency for all models in both the test set (**Figure 6A**) and external validation set (**Figure 6B**). DCA curves showed that, both in the test set (**Figure 7A**) and external validation set (**Figure 7B**), all models achieved higher net benefit than the “no intervention” and “all intervention” lines across a large range of thresholds, indicating their potential clinical utility. Although the NN showed the highest AUC in the test set in the multimodel comparison, its discriminative abilities and generalizability in the external validation cohort were comparable to those of LR and SVM. While the NN offered a higher net clinical benefit across the majority of threshold ranges in the test set, the decision curve analysis also showed that this advantage significantly decreased in the external validation cohort, where its net-benefit curve overlapped with LR’s across the majority of thresholds. In general, the LR model performed more consistently across various datasets.

**Table 4:** Performance evaluation metrics of models constructed by each algorithm.

Dataset	Model name	Accuracy	Sensitivity	Specificity	Precision	F1 score	Brier score
Test	Logistic	0.816	0.831	0.786	0.885	0.857	0.134
	SVM	0.800	0.819	0.762	0.872	0.845	0.145
	GBM	0.800	0.952	0.500	0.790	0.863	0.159
	NeuralNetwork	0.848	0.904	0.738	0.872	0.888	0.123
	XGBoost	0.808	0.892	0.643	0.831	0.860	0.207
	KNN	0.792	0.843	0.690	0.843	0.843	0.153
	AdaBoost	0.704	0.687	0.738	0.838	0.755	0.174
	LightGBM	0.728	0.807	0.571	0.788	0.798	0.209
	CatBoost	0.752	0.831	0.595	0.802	0.817	0.208
Valid	Logistic	0.808	0.890	0.667	0.820	0.854	0.160
	SVM	0.800	0.829	0.750	0.850	0.840	0.158
	GBM	0.769	0.805	0.708	0.825	0.815	0.165
	NeuralNetwork	0.831	0.963	0.604	0.806	0.878	0.151
	XGBoost	0.785	0.927	0.542	0.776	0.844	0.211
	KNN	0.785	0.902	0.583	0.787	0.841	0.177
	AdaBoost	0.723	0.720	0.729	0.819	0.766	0.199
	LightGBM	0.808	0.915	0.625	0.806	0.857	0.227
	CatBoost	0.700	0.683	0.729	0.812	0.742	0.227

### 3.5 Model interpretation and application

**Figure 8:**
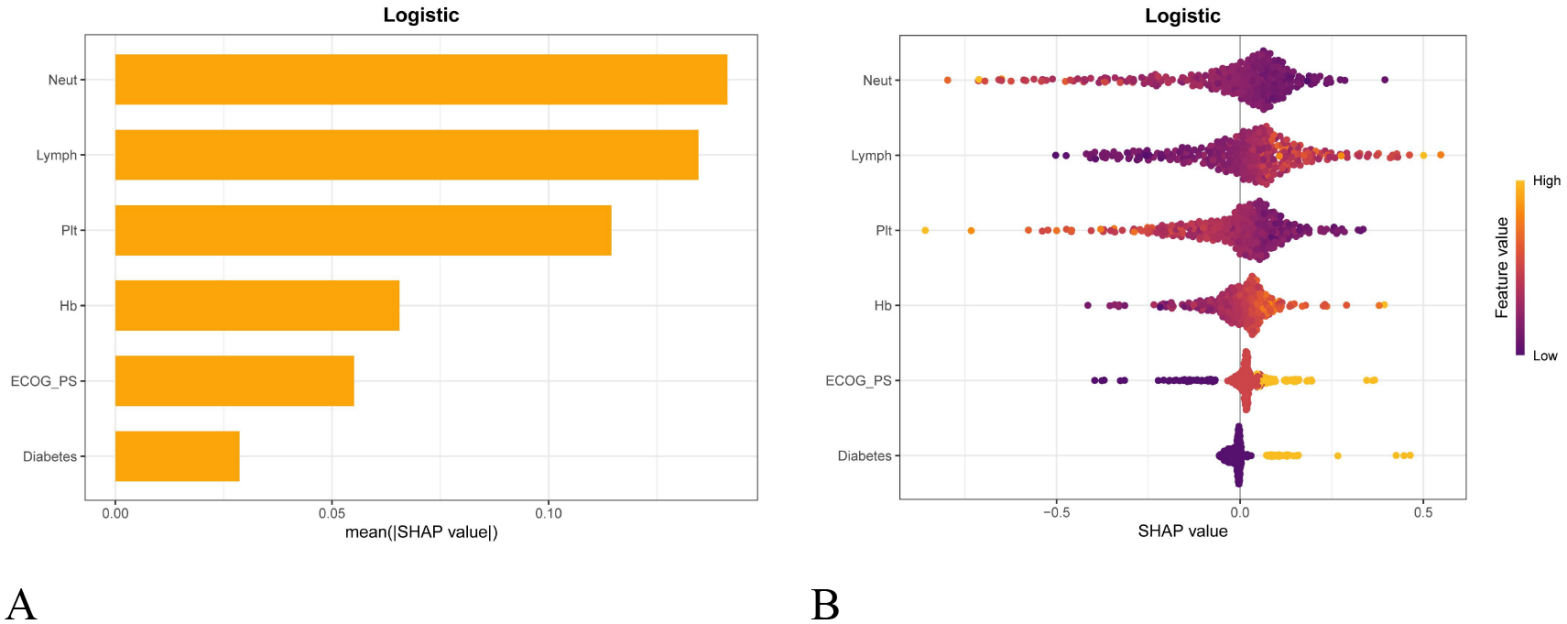
SHAP analysis of the LR model: **(A)** Mean absolute SHAP values showing global feature importance. The x-axis represents the mean absolute SHAP value, with larger values indicating a greater overall impact of the corresponding feature on the model predictions. The features were ranked in descending order of importance as follows: Neut, Lymph, Plt, Hb, ECOG PS, and diabetes history. **(B)** SHAP swarm plot illustrating the direction and magnitude of feature contributions. The y-axis lists the features included in the model, ranked according to their importance, and the x-axis represents the corresponding SHAP values. Each dot represents the contribution of a given feature to the model prediction for an individual sample. The color of the dots ranges from purple to yellow, indicating low to high feature values, respectively. For example, as the lymphocyte count increases (from purple to yellow), the SHAP value shifts from negative to positive, suggesting that lymphocyte count is positively associated with the outcome.

**Figure 9:**
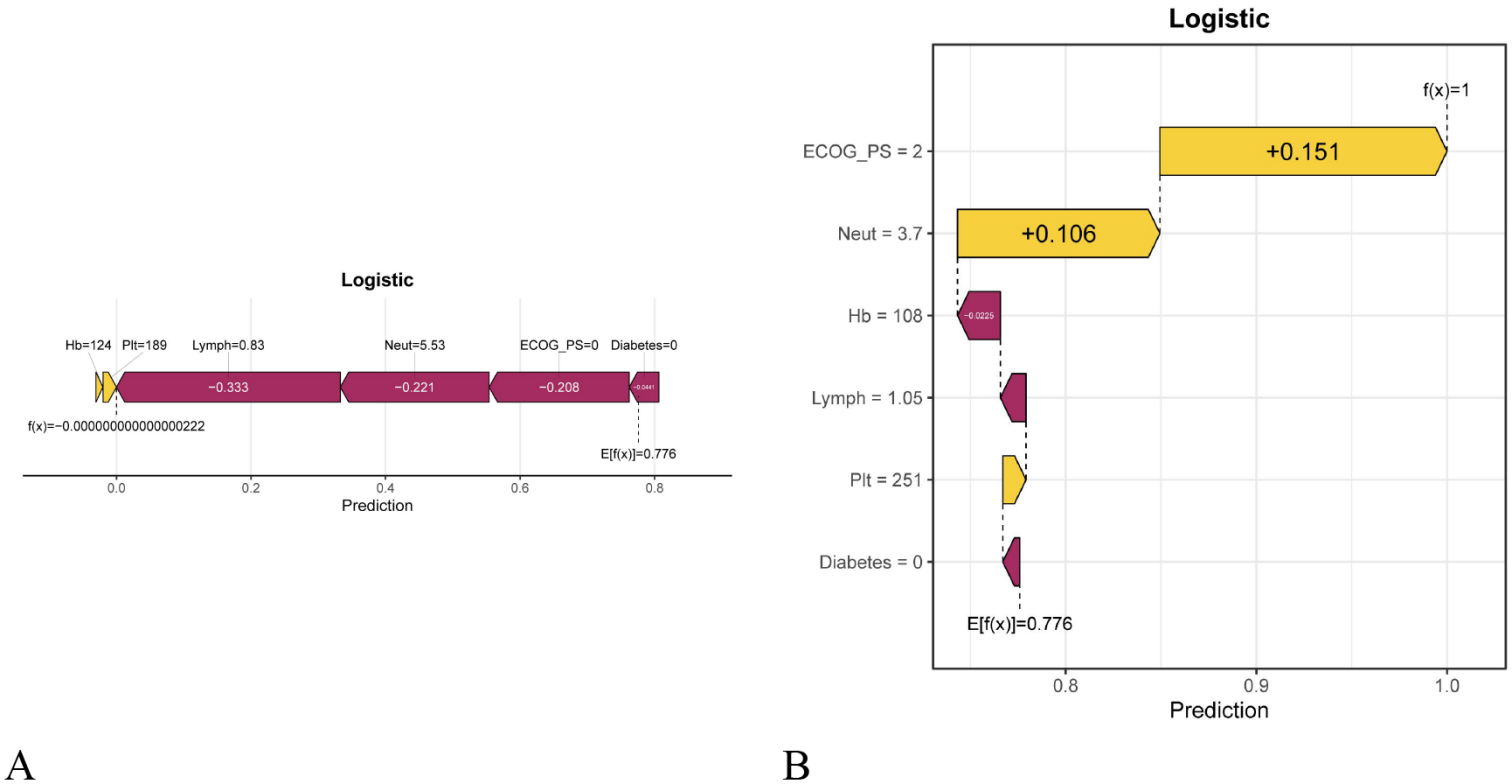
Patient-level SHAP interpretation of the LR model: **(A)** The SHAP force plot is used to illustrate the contribution of different features to the prediction outcome for an individual patient. The direction of the arrows indicates the direction of each feature’s effect. Specifically, yellow arrows pointing to the right indicate that the corresponding feature makes a positive contribution to the risk of irAEs, whereas purple arrows pointing to the left indicate that the feature makes a negative contribution to the risk of irAEs. The length of the arrows helps visualize the magnitude of the predictive effect, with longer arrows indicating a greater effect. E[f(x)] represents the average model prediction, and f(x) represents the individual prediction, thereby intuitively reflecting how different features influence the predicted outcome for a specific patient. **(B)** The SHAP waterfall plot starts from the model’s baseline prediction, E[f(x)], and sequentially adds or subtracts the contribution of each feature (SHAP value) according to its effect on the prediction, ultimately reaching the final prediction for the sample, f(x). Yellow bars extending to the right indicate positive contributions of the corresponding features, whereas purple bars extending to the left indicate negative contributions. The numerical value shown next to each feature (e.g., + 0.151) represents the specific contribution of that feature to the model prediction.

This study used SHAP to interpret the prediction results of the LR model. As shown in **Figure 8A**, Neut was the most important global feature, followed by Lymph, Plt, Hb, ECOG PS, and diabetes history. **Figure 8B** further illustrates the direction of each feature’s influence on irAEs risk, with each point representing a sample and the color indicating the magnitude of the feature value (yellow for high values and purple for low values). The results showed that higher Neut and Plt levels were associated with a lower risk of irAEs, whereas higher Lymph, ECOG PS, and Hb values, as well as a history of diabetes, were associated with an increased risk. To provide patient-level interpretability, SHAP force and waterfall plots were generated for two representative cases (**Figures 9A, B**). Features that increased the predicted risk are shown in yellow, while those that reduced the risk are shown in purple. The length of each arrow reflects the magnitude of the effect, and its orientation indicates the direction of the feature’s influence. Furthermore, to enhance clinical decision-making and practice, we developed an intuitive online web application tool based on the LR model (https://lingchun.shinyapps.io/web123/) to facilitate the assessment of irAEs risk (See **Supplementary Figure 3**).

## 4 Discussion

In this study, we utilized nine machine learning algorithms to construct models for predicting the risk of irAEs in advanced NSCLC patients receiving ICI therapy. The results showed that the NN model attained the superior discriminative performance in the test set (AUC = 0.881) and yielded greater clinical net benefit across the most threshold probabilities. However, this advantage was significantly diminished in the independent temporal external validation cohort, where its discriminative capability became similar to that of more straightforward models like LR and SVM. Furthermore, the decision curves confirmed that the NN retained only marginal benefit within a limited range of thresholds, with overall differences among models remaining small. These findings indicate that although complex models can capture more intricate nonlinear relationships within internal datasets, their additional complexity does not translate into substantial clinical benefit. Taking into account discrimination, calibration, stability, and the practical need for model transparency, we chose LR for translational application and developed a user-friendly web-based prediction tool to assist clinicians in efficiently identifying patients at increased risk of irAEs and supporting more individualized management strategies.

The application of ICIs has provided significant clinical benefits for cancer patients (29, 30). However, the risk of immune therapy-related toxicities that comes with it cannot be ignored (31). In this study, out of 550 advanced NSCLC patients receiving ICI treatment, 361 cases (65.6%) developed irAEs. In our cohort, the incidence of high-grade adverse events (grade ≥ 3) was 10% (55/550). Consistent with these findings, Chen et al. previously reported a 58.5% overall incidence of irAEs in a cohort of 301 NSCLC patients, among whom 22.3% discontinued therapy as a result of adverse events (32). A Phase III open-label trial (N = 305) demonstrated that pembrolizumab significantly improved progression-free survival (PFS) and overall survival (OS) relative to chemotherapy. However, the incidence of any-grade irAEs in the pembrolizumab group was as reached as 73.4% (33). Therefore, actively monitoring and predicting the risk of irAEs may help mitigate their negative impact on overall treatment efficacy.

In recent years, identifying patients at high risk of irAEs has attracted considerable attention from researchers. Gao et al. (34) conducted a retrospective analysis of 484 NSCLC patients who received ICI treatment from January 2019 to December 2021. They used univariate and multivariate logistic regression to identify independent risk factors for irAEs and developed a nomogram based on these factors. The model achieved AUCs of 0.851 and 0.779 in the training and internal test sets, respectively, demonstrating good discriminative capability but lacking effective validation in an external cohort. Xu et al. (35) carried out a multicenter retrospective study involving 1,197 NSCLC patients from different hospitals. In the context of sintilimab monotherapy, they similarly developed a nomogram to predict irAE risk and performed external validation. The model achieved AUC values ranging from 0.754 to 0.835 for predicting 1- to 2-year irAEs, reflecting favorable discriminatory ability. Compared with these earlier studies, our research offers several methodological and clinical distinctions. First, we incorporated the real-world immunotherapy patterns commonly observed in clinical practice, including various commonly used PD-1/PD-L1 inhibitors and treatment strategies, thereby ensuring that the model is broadly applicable and more representative of actual clinical scenarios. Second, prior to model development, we used LASSO regression for feature selection, which reduced model complexity, lowered the risk of overfitting, and improved the overall stability of the model. Subsequently, we compared nine machine learning approaches, covering both linear and nonlinear techniques. Rather than assuming the superiority of any single algorithm, we evaluated them from multiple perspectives, including AUC, accuracy, sensitivity, specificity, precision, F1 score, Brier score, and clinical net benefit. Such a comprehensive comparison minimized the potential biases associated with relying on a single algorithm and provided stronger evidence for selecting the most appropriate model. Additionally, we validated the models using a temporally independent external cohort, further ensuring their generalizability. Based on these comparisons, LR was ultimately selected as the final predictive model. In the test set and external validation cohort, the AUC values (95% CI) were 0.855 (0.777–0.934) and 0.801 (0.712–0.890), respectively, pointing to solid model discrimination. The F1 scores were 0.857 and 0.854, indicating high accuracy and stability, while the Brier scores of 0.134 and 0.160 suggested satisfactory calibration performance. Finally, we examined the contribution of each predictor in the LR model using SHAP and deployed the model as an online web-based calculator, allowing clinicians to access individualized irAE risk estimates and facilitating practical use at the bedside.

In this study, we identified six variables: Neut, Lymph, Plt, Hb, a history of diabetes, and ECOG PS ≥ 2 as being significantly linked to the risk of irAEs in patients with advanced NSCLC receiving ICI therapy. A growing body of evidence suggests that hematological parameters play an important role in predicting irAEs in cancer patients undergoing ICIs treatment. Studies by Moey and Peng (36, 37) showed that a lower baseline neutrophil-to-lymphocyte ratio (NLR) was closely related to an increased likelihood risk of irAEs. The clinical data analysis by Michailidou et al. on 470 cancer patients not only supported the predictive role of a low baseline (NLR ≤ 5.3) but also further revealed that lower baseline platelet-to-lymphocyte ratio (PLR ≤ 534) and monocyte-to-lymphocyte ratio (MLR ≤ 0.73), as well as high baseline lymphocyte count (> 2.6 k/ul), monocyte count (> 0.29 k/ul), and platelet count (> 145 k/ul), were all associated with the occurrence of irAEs (38). Consistent with these findings, univariate analysis in both the total development set and training set of this study revealed that lower NLR, PLR, and higher Lymph levels, were significantly related to the occurrence of irAEs (P < 0.05). However, in contrast to Michailidou’s report that higher baseline platelet counts (> 145 k/ul) and monocyte count (> 0.29 k/ul) increase the risk of irAEs, this study found that lower baseline platelet count and neutrophil count were significantly tied to irAEs in univariate analysis of both the total development set and training set, whereas monocyte count did not reach statistical significance (P = 0.082 and P = 0.128, respectively). These discrepancies may stem from heterogeneity in the study populations. The study by Michailidou included patients with various cancer types, while this study specifically focused on NSCLC patients. It should be particularly noted that although univariate analysis in this study shows an association between low baseline platelet count and the risk of irAEs, the median platelet levels in both the overall development and training sets were 199 k/ul and 209 k/ul, respectively, which actually fall within the “high-risk” ranges (> 145 k/ul) defined in Michailidou’s study. Future research or clinical interpretations should fully consider the differences in population characteristics and disease background. It is noteworthy that ratio metrics such as NLR, PLR, and LMR demonstrated significant association with irAEs in univariate analysis, yet none were incorporated into the final predictive model during subsequent multivariate analysis. The core reason lies in the high statistical multicollinearity between these ratio metrics and their constituent baseline blood cell counts (Neut, Lymph, Plt, Mono). When these underlying blood cell counts were simultaneously included in the model, the information they contained largely overlapped with the predictive signals provided by the ratio indicators. Consequently, the independent predictive value of the latter became statistically insignificant, leading to their exclusion from the model. This suggests that, within the multivariate predictive model constructed for this study cohort, the direct measurements of underlying blood cell counts possess stronger independent predictive capability than the ratios calculated from them.

At the same time, this study found that patients with an ECOG PS ≥ 2 had a higher risk of developing irAEs (OR = 5.181, P = 0.023), which is consistent with the findings of Ksienski, Suazo-Zepeda, and others (39, 40). The ECOG PS score typically reflects a patient’s physical condition and overall health. A higher score suggests poorer performance status, which may result in lower tolerance to immunotherapy and thus affect the occurrence of irAEs. Similarly, in this study, patients with a history of diabetes had a 5.054 times higher risk of developing irAEs compared to those without a history of diabetes (P = 0.034), suggesting that clinical use of immunotherapy drugs in NSCLC patients with diabetes should be approached with greater caution. Diabetes may influence ICI tolerability through mechanisms related to metabolic disturbances, chronic inflammation, or changes in the immune microenvironment. However, due to the retrospective nature of this study, metabolic indicators such as HbA1c and complete lipid profiles were missing in a substantial proportion of patients, which prevented us from conducting a more detailed analysis of their potential mechanistic roles. In future work, we plan to conduct prospective studies to further explore the potential significance of metabolic status in predicting irAE risk. Previous studies (41) have indicated that high baseline Hb levels (OR = 1.024, P = 0.040) are also associated with the occurrence of irAEs, and this study supports that conclusion. The specific mechanism remains unclear and requires further research in the future.

There are still some limitations in our study. First, as a retrospective study, potential biases cannot be completely avoided, even though we used strict inclusion and exclusion criteria during patient selection. Second, the immune checkpoint inhibitors and immunotherapy regimens included in this study primarily represent the most widely used treatment approaches in current clinical practice, such as the PD-1 inhibitor sintilimab, the PD-L1 inhibitor durvalumab, and immunotherapy in combination with chemotherapy. Within this treatment framework, neither the type of immune checkpoint inhibitor nor the specific immunotherapy regimen showed an obvious association with the occurrence of irAEs in our cohort. As newer agents are introduced and more complex therapeutic combinations become routine, and as analyses are extended to larger patient groups, different associations may emerge. Such evolving treatment paradigms may influence risk factor identification and model performance. Finally, although we included a time-separated validation cohort to strengthen the reliability of our findings, all data were obtained from a single center, which may limit the applicability of the model to other populations. Therefore, in future research, we plan to incorporate newly developed ICIs and more diverse combination therapies, and to conduct large-scale, multicenter, prospective cohort studies to better clarify the relationship between immunotherapy strategies and irAE risk, thereby further improving the stability and generalizability of the model.

## 5 Conclusion

In summary, we employed machine learning to develop a novel predictive model for forecasting the occurrence of irAEs in patients with advanced NSCLC following different immunotherapy regimens. This model underwent external validation using time-series cohort data, demonstrating high predictive accuracy, robust stability, and strong generalisability. It offers a scientifically grounded basis for clinical decision-making, aiding clinicians in formulating personalised treatment plans within resource-constrained settings. Future research should further refine this model and explore its application across multicentre settings and larger sample sizes to enhance its clinical utility.

## Data Availability

The original contributions presented in the study are included in the article/**Supplementary Material**. Further inquiries can be directed to the corresponding author.
