# Supplementary material for: Development of an online prediction tool for immunotherapy-related adverse events in patients with advanced NSCLC based on machine learning and temporal validation
Source: Front Oncol. 2026 May 1;16:1711801. doi: 10.3389/fonc.2026.1711801 (PMC13175838; doi:10.3389/fonc.2026.1711801)
Supplement: Supplementary file 1 [file DataSheet1.zip › Supplementary_Material.pdf]

Supplementary Material

Variable	Training Set	Test Set	Validation Set
Height(cm)	3(1.0)	2(1.6)	0
Weight	3(1.0)	2(1.6)	0
BMI	3(1.0)	2(1.6)	0
PT(s)	26(8.8)	11(8.8)	1(0.8)
APTT(s)	26(8.8)	11(8.8)	1(0.8)
TT(s)	26(8.8)	11(8.8)	1(0.8)
D-dimer(μg/ml)	26(8.8)	11(8.8)	1(0.8)
LDH(U/L)	46(15.6)	10(8.0)	14(10.8)
CEA(ng/ml)	29(9.8)	11(8.8)	6(4.6)
NSE(ng/ml)	29(9.8)	11(8.8)	6(4.6)
CYFRA21-1(ng/ml)	29(9.8)	11(8.8)	6(4.6)
ALP(U/L)	32(10.8)	9(7.2)	25(19.2)
CREA(μmol/L)	9(3.1)	0	0
TC(mmol/L)	35(11.9)	16(12.8)	6(4.6)
TG(mmol/L)	35(11.9)	16(12.8)	6(4.6)
K(mmol/L)	15(5.1)	7(5.6)	4(3.1)
Na(mmol/L)	15(5.1)	7(5.6)	4(3.1)
Ca(mmol/L)	15(5.1)	7(5.6)	4(3.1)

Supplementary Table 1. Distribution of Missing Data in Training, Test, and Validation Sets.

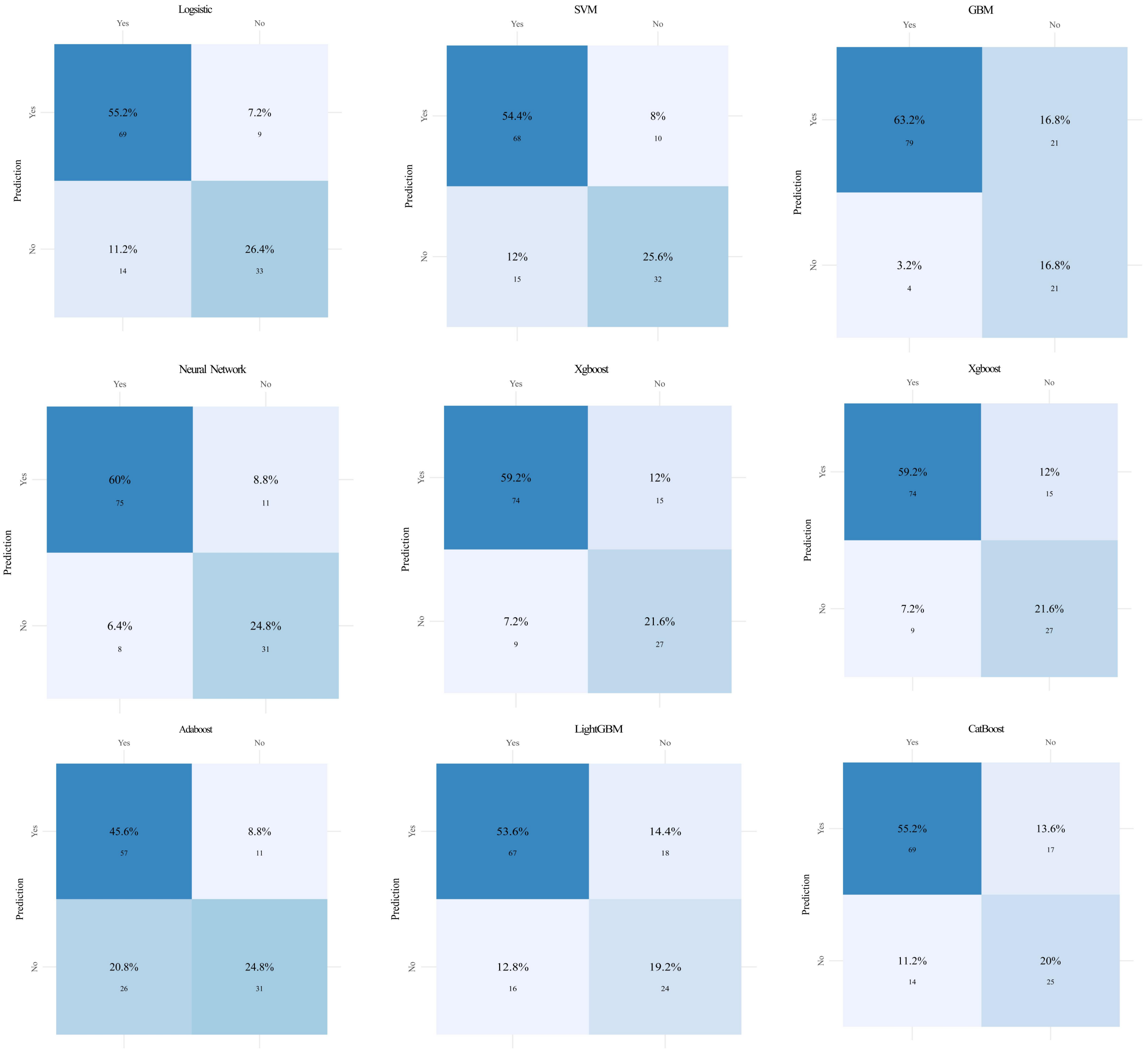

Supplementary Figure 1. Confusion matrix for the test set.

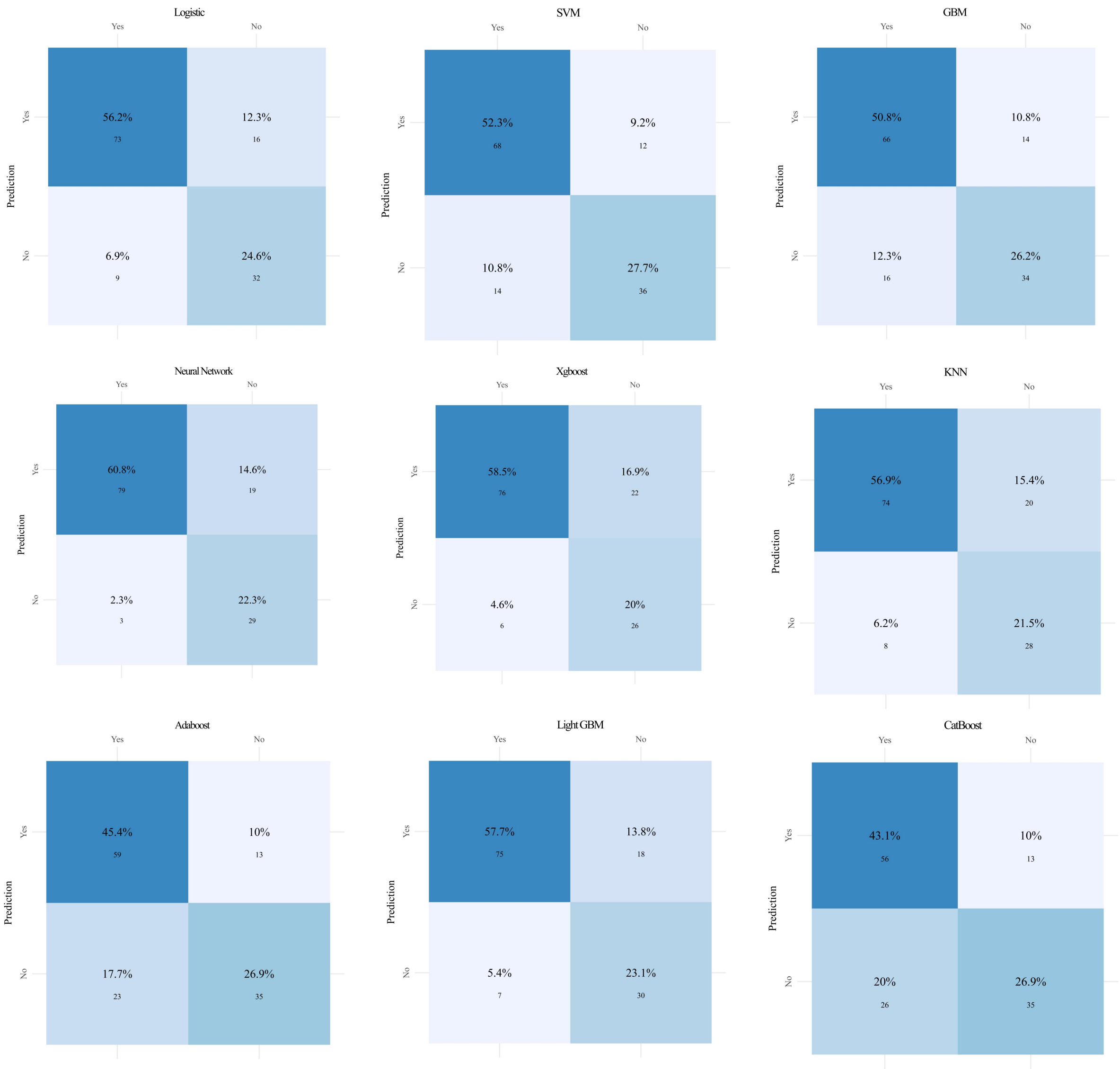

Supplementary Figure 2. Confusion matrix for the external validation set.

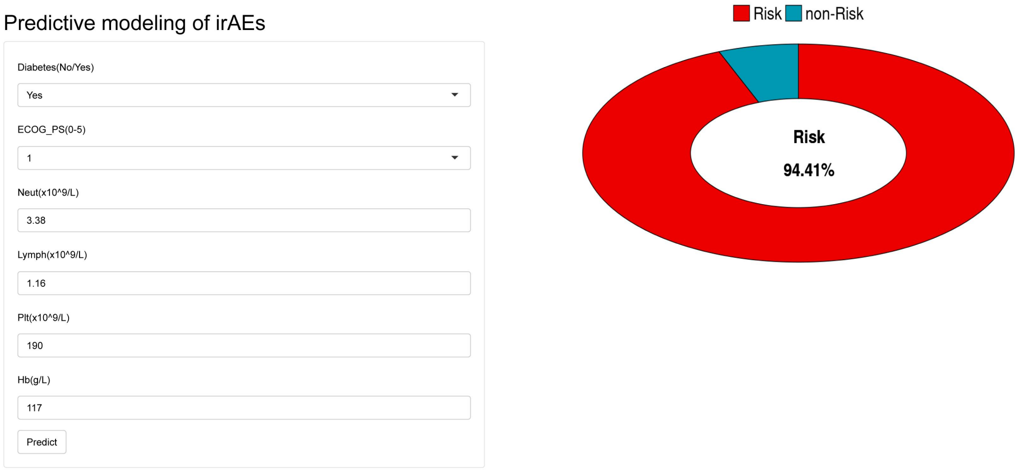

Supplementary Figure 3. An online calculator derived from the logistic regression model.
